# Attenuation of *Helicobacter pylori*-induced gastric inflammation by prior *cag*^−^ strain (AM1) infection in C57BL/6 mice

**DOI:** 10.1186/s13099-017-0161-5

**Published:** 2017-03-09

**Authors:** Nillu Ghosh, Prachetash Ghosh, Kousik Kesh, Asish K. Mukhopadhyay, Snehasikta Swarnakar

**Affiliations:** 1 Cancer Biology and Inflammatory Disorder Division, Indian Institute of Chemical Biology, 4, Raja S.C. Mullik Road, Jadavpur, Kolkata, 700032 India; 2 National Institute of Cholera and Enteric Diseases, Kolkata, India

**Keywords:** *Helicobacter pylori*, *Cag* pathogenicity island, Coinfection, Gastric ulcer, MMP, Cytokine, Inflammation, Immunosuppression

## Abstract

**Background:**

*Helicobacter pylori,* colonize in stomach of ~50% of the world population. *cag* pathogenicity Island of *H. pylori* is one of the important virulent factors that attributed to gastric inflammation. Coinfection with *H. pylori* strain with different genetic makeup alters the degree of pathogenicity and susceptibility towards antibiotics. The present study investigates host immunomodulatory effects of *H. pylori* infection by both *cag* ^+^ strain (SS1) and *cag* ^−^ strain (AM1). C57BL/6 mice were infected with AM1 or SS1 strain as well as AM1 followed by SS1 (AM1/SS1) and vice versa.

**Results:**

Mice infected with AM1/SS1 strain exhibited less gastric inflammation and reduced proMMP9 and proMMP3 activities in gastric tissues as compared to SS1/SS1 and SS1/AM1 infected groups. The expression of both MMP9 and MMP3 followed similar trend like activity in infected tissues. Both Th1 and Th17 responses were induced by SS1 strain more profoundly than AM1 strain infection which induced solely Th1 response in spleen and gastric tissues. Moreover, IFN-γ, TNF-α, IL-1β and IL-12 were significantly downregulated in mice spleen and gastric tissues infected by AM1/SS1 compared to SS1/SS1 but not with SS1/AM1 coinfection. Surprisingly, IL-17 level was dampened significantly in AM1/SS1 compared to SS1/AM1 coinfected groups. Furthermore, number of Foxp3^+^ T-regulatory (Treg) cells and immunosuppressive cytokines like IL-10 and TGF-β were reduced in AM1/SS1 compared to SS1/SS1 and SS1/AM1 coinfected mice gastric tissues.

**Conclusions:**

These data suggested that prior *H. pylori cag* ^−^ strain infection attenuated the severity of gastric pathology induced by subsequent *cag* ^+^ strain in C57BL/6 mice. Prior AM1 infection induced Th1 cytokine IFN-γ, which reduced the Th17 response induced by subsequent SS1 infection. The reduced gastritis in AM1/SS1-infected mice might also be due to enrichment of AM1- primed Treg cells in the gastric compartment which inhibit Th1 and Th17 responses to subsequent SS1 infection. In summary, prior infection by non-virulent *H. pylori* strain (AM1) causes reduction of subsequent virulent strain (SS1) infection by regulation of inflammatory cytokines and MMPs expression.

## Background

*Helicobacter pylori*, a class I carcinogen inhabit in the stomach of approximately 50% of the human population while only 10–15% population either develop chronic gastritis or gastric adenocarcinoma or gastric mucosa-associated lymphoid tissue lymphoma [1–4]. The underlying mechanisms governing the clinical outcome of *H. pylori* infection are poorly understood. However, accumulated evidences suggested that differences in host immune responses, environmental factors as well as the virulence properties of *H. pylori* strains may play important roles in determining the disease outcome. The most prominent *H. pylori* virulence-associated determinant is the *cag* pathogenicity island (PAI). It is a 40-kb genome segment that encodes the immunodominant protein *cag*A and type IV secretion system, which serve to transfer the bacterial *cag*A protein and other soluble factors such as peptidoglycans, to the cytoplasm of the host cells, known to play a key role in disease manifestation [5–7]. Strains harboring the *cag* PAI have been associated with more severe inflammatory responses than that induced by *cag* ^−^ strains [6, 8, 9]. *H. pylori* specific host T cell response is predominantly a CD4+ T cell response polarized towards a T helper1 (Th1) phenotype [10, 11]. *H. pylori* induced inflammation are associated with the production of pro-inflammatory cytokines and appear to be triggered partly by genes located within the *cag* PAI [6, 8, 12]. The gastric mucosal levels of the proinflammatory cytokine IL-1β, IL-6, IL-8 and TNF-α were increased in *H. pylori* infected subjects [13]. Earlier studies revealed that *H. pylori* infection is also associated with a marked increased in cytokine IL-17 secretion from Th17 cells [14]. Involvement of IL-17 has also been reported in various other chronic inflammatory conditions such as rheumatoid arthritis and multiple sclerosis [15, 16]. Recently, Shi et al. suggested that *H. pylori* infection induced a mixed Th1/Th17 response [17]. In addition, *cag*A and type IV secretion system are required for the induction of IL-17 responses in *H. pylori* infection [18]. Secretion of IL-17 led to induction of other inflammatory molecules required for the establishment of chronic inflammation [19].

MMPs are a family of zinc dependent endopeptidases that play a crucial role in various pathological conditions including gastric ulcer [20–22]. The activities of MMPs are regulated by their inhibitors (TIMPs), while their expressions are modulated by cytokines, growth factors, tumor promoters and transcription factors [20, 23, 24]. Gelatinases B (MMP9) and stromelysin-1 (MMP3) are the two major inflammatory contributors of gastric pathology, collectively cleave a large array of matrix proteins [25]. Accumulated evidences suggested that *H. pylori* induced gastric inflammation with the upregulation of MMP9 and MMP3 in vivo [26]. MMPs are either directly or indirectly produced by gastric epithelial cells via cytokine mediated cell signaling pathways [24].

The gastrointestinal tract of human are colonized by various microorganisms which can be either commensalistic or pathogenic to human [27]. The interplay among those organisms can lead to either attenuation or promotion of infection-induced pathology. For instance, C57BL/6 mice coinfection with a natural murine nematode parasite *Heligmosomoides polygyrus*, attenuated gastric pathology induced by *H. felis* [28]. Attenuation of gastric pathology was associated with reduced expression of proinflammatory Th1 cytokine as well as with increased Th2 cytokine levels. Interaction between different bacterial species also determines diseases severity. Recently it has been documented that *H. pylori* infection attenuated *Salmonella enterica* serovar Typhimurium-induced colitis in C57BL/6 mice; this protective effect was associated with downregulation of the cecal Th17 response to *S. typhimurium* [29]. Even severity of the *H. pylori* induced gastric pathology is also modulated by coinfection with other *Helicobacter* species. Coinfection of enterohepatic Helicobacter species (EHS), *Helicobacter muridarum* along with *H. pylori* attenuated the *H. pylori* induced gastric pathology in C57BL/6 mice [30]. Moreover, coinfection of another EHS, *Helicobacter hepaticus* with *H. pylori* lead to more severe gastritis as well as increased production of IL-17 cytokine [30]. Interestingly, it has also been reported that the interactions between different strains of *H. pylori* also modulate gastric inflammation status [31].

Surprisingly, in some African countries having lower economic status show high rate of *H. pylori* infection but low level of gastric carcinoma incidence, widely known as African Enigma [32, 33]. The reasons could be associated with diet, infection with other endemic parasites and degree of pathogenicity of different *H. pylori* strains. We hypothesized that coinfection with a non pathogenic strain may provide protection against further infection of a virulent strain. The effect of coinfection on gastric inflammation, with different strains of *H. pylori* with or without *cag* Pathogenicity Island, has not been systematically studied yet. To investigate that, we established a coinfection in C57BL/6 mice using both *cag* ^−^ and *cag* ^+^ strains of *H. pylori* and measured the gastric inflammatory pathways. We address whether prior *cag* ^−^ strain infection alleviate gastric damage induced by *cag* ^+^ *H. pylori* strain coinfection and the underlying host immunomodulatory mechanisms thereon. Here we for the first time documents that prior infection with *cag* ^−^ *H. pylori* strain dampens the disease severity for further *cag* ^+^ coinfection.

## Results

### Prior *cag*^−^*H. pylori* infection dampens gastric inflammation due to *cag*^+^*H. pylori* infection

**Fig. 1:**
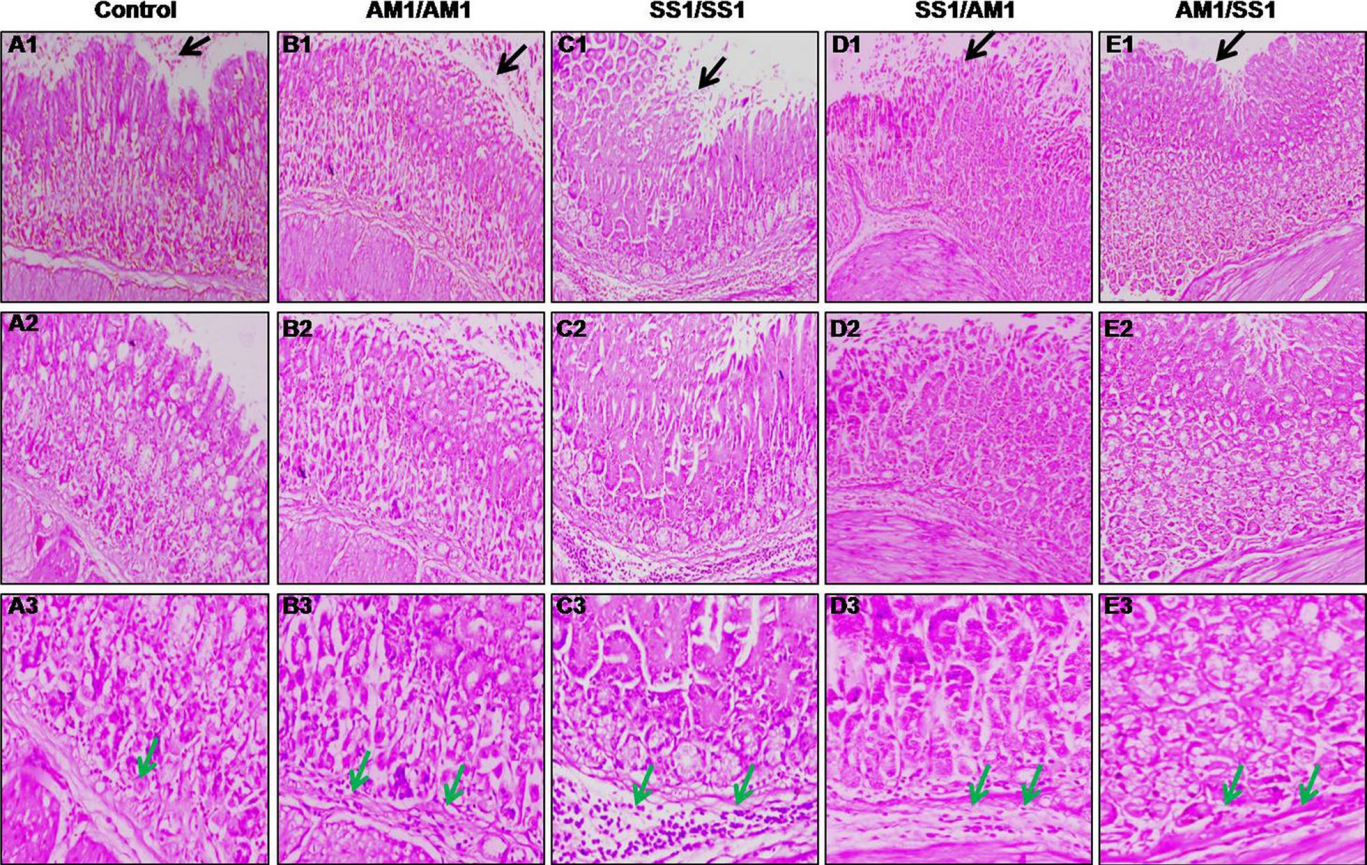
Histology of mouse gastric tissues infected with *H. pylori*. Different groups of mice were intragastrically inoculated with SS1 (*cag* ^+^) and AM1 *(cag* ^−^) strains alone or coinfected with both strains of *H. pylori* (SS1 followed by AM1, AM1 followed by SS1). Control mice were fed with PBS and kept separately under the same conditions. Mice were sacrificed and gastric tissue sections were processed for histological analysis. Histological appearances of control (*A1*), AM1/AM1 infected (*B1*), SS1/SS1 infected (*C1*), SS1/AM1-coinfected (*D1*), AM1/SS1-coinfected (*E1*) gastric tissues stained with hematoxylin and eosin and were observed at 10× magnification. While *A2*, *B2*, *C2*, *D2* and *E2* represent 20× magnification and *A3*, *B3*, *C3*, *D3* and *E3* represent 40× magnification of control, AM1/AM1, SS1/SS1, SS1/AM1, AM1/SS1-infected tissues. Gastric mucosal epithelium and inflammatory cells are shown by *black arrows* and *green arrows*, respectively

We previously reported that both *cag* ^+^ strains (SS1) and *cag* ^−^ strain (AM1) has the capacity to induce gastric inflammation, although the severity of damage was more pronounced in SS1 infection in C57BL/6 mice [26]. In present study, we investigated the coinfection of different strains of *H. pylori* in different combination on disease burden of *H. pylori* induced gastric pathology in C57BL/6 mice. Different groups of mice were inoculated with vehicle for control, SS1 and boosted with SS1 strain, inoculated with AM1 boosted with AM1 strain, SS1 and boosted with AM1 strain and AM1 and boosted with SS1 strain of *H. pylori* separately and were sacrificed at day 10 post infection. Histological examination of mouse gastric tissues revealed that *H. pylori* infection in any combination of strains caused inflammation in gastric pit cells along with disruption in submucosa and muscularis mucosa compared with control (Fig. 1). Glandular atrophy and infiltration of inflammatory cells, mostly lymphocytes, were also detected in the gastric tissues of all infected mice groups. However, mice inoculated with AM1 followed by SS1 strain exhibited significantly lower level of gastric inflammation, glandular atrophy, surface epithelial eruption and decreased infiltration of inflammatory cells compared to SS1/SS1 infected and SS1/AM1 coinfected mice. However, no significant difference in the gastric lesions in AM1/AM1 and AM1/SS1 coinfected mice were detected (Fig. 1b, e). Therefore, these results suggested that earlier *cag* ^−^ *H. pylori* strain infection significantly abrogated the severity of gastric inflammation induced by further *H. pylori cag* ^+^ strain infection.

### The activity and expression of MMP9 and 3 were upregulated with increased severity of gastric lesion

**Fig. 2:**
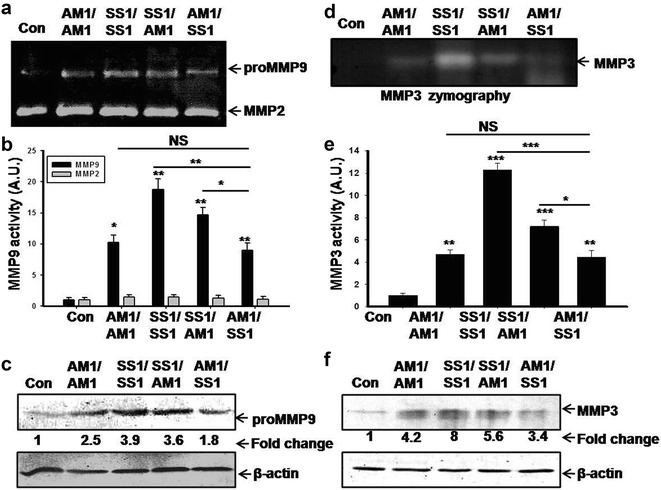
Effect of *H. pylori* infection and coinfection on activity and expression of MMPs. Different strains of *H. pylori* (AM1 or SS1) were orally fed separately or coinfected (AM1/SS1or SS1/AM1) to four groups of mice and they were sacrificed on day 10 post infection. Control mice were fed with PBS and kept separately under same conditions. The activities of MMP2 and 9 in mouse gastric tissue extracts were measured by gelatin zymography (**a**). Histographic representations of gelatinolytic activities as measured by lab image densitometry (**b**). Values were from the above zymograms and three other zymograms from independent experiments. Expression of MMP9 was measured by Western blotting analysis (**c**). Equal amount of tissue extracts (120 μg) of control and infected mice were used and probed with polyclonal anti-MMP9 and monoclonal anti-β actin antibody. The activity and expression of MMP3 in mouse gastric tissue extracts were measured by casein zymography and Western blot. Representative blots showing the activity and expression MMP3 (**d**, **f**). β-actin served as loading control. Histographic representations of MMP3 activity and expressions in control and *H. pylori* infected gastric tissues (**e**) from the *above blots* and two other representative blots from independent experiments in each case. *Error bars* ±SEM ***P < 0.001; **P < 0.01; *P < 0.05; NS, P = not significant versus control

Aberrant ECM remodeling is a prerequisite event in gastric ulcer development. MMP2 and 9 are the two most potent enzymes involved in the ECM remodeling. Hence, we measured and compared the activity and expression of MMP2 and 9 by gelatin zymography and Western blotting respectively in *H. pylori* infected mice gastric tissue extracts. We found that there is a significant upregulation of MMP9 activity and expression in *H. pylori* infected gastric tissues compared with control (Fig. 2a–c). Highest level of activity and expression of MMP9 were obtained in SS1/SS1 infected gastric tissues. However, interestingly AM1/SS1 coinfected mice showed decreased level of MMP9 expression and activity compared to SS1/SS1 infected and SS1/AM1 coinfected gastric tissues. In between coinfected groups, MMP9 expression and activity were detected higher in SS1/AM1 in compared to AM1/SS1 coinfected group. We also measured the activity and expression of another potent ECM degrading enzyme MMP3 in *H. pylori* infected and control mice gastric tissues. Like MMP9 similar trends of activity and expression pattern of MMP3 were detected (Fig. 2d–f). Highest level of MMP3 activity and expression were detected in SS1 infected group. Notably, lower level of MMP3 expression and activity were also detected in AM1/SS1 coinfected group.

**Fig. 3:**
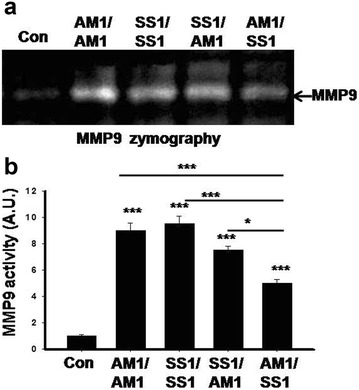
Serum MMP9 level reduced in AM1/SS1 coinfected mice. Different groups of mice were orally fed with either SS1 or AM1 strains alone or coinfected with both strains of *H. pylori* (SS1 followed by AM1, AM1 followed by SS1). Control mice were fed with PBS and kept separately under the same conditions. Activity of MMP9 in mice serum was assayed by gelatin zymography (**a**). Equal volumes of serum were loaded in each lane for gelatin zymography. Histographic representation of gelatinolytic activity as measured by lab image densitometry (**b**). Data were represented as mean ± SD from three independent sets of experiment. *Error bars* ±SEM ***P < 0.001; *P < 0.05 NS, P = not significant versus control

To examine the effect of *H. pylori* infection on systemic level of MMP9 and 2, we measured the activity of MMP9 in infected mice serum. Figure 3 shows that *H. pylori* infection also increases activity of proMMP9 in mice serum. In addition, AM1/SS1 coinfected mice show decreased level of serum MMP9 activity compared to SS1 infected and SS1/AM1 coinfected groups.

### Cytokine expression pattern differed in different combination of *H. pylori* infection

**Fig. 4:**
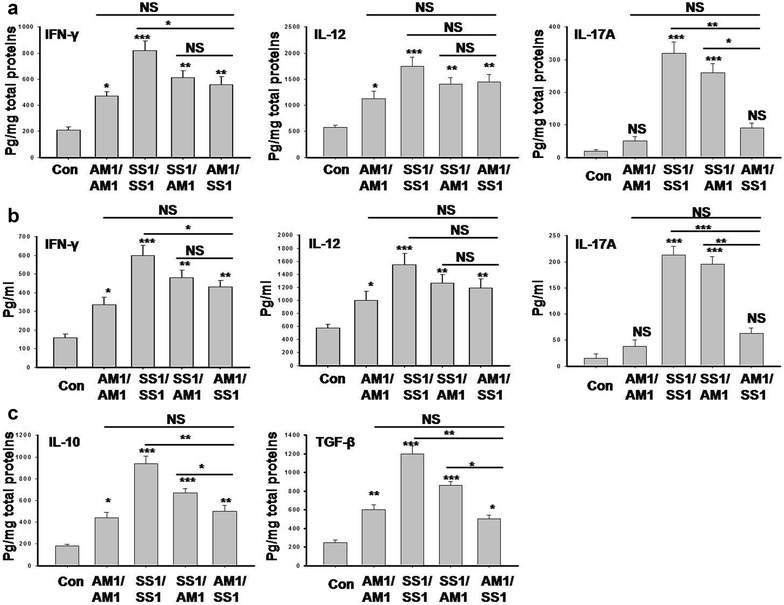
Level of cytokines in mouse gastric tissues and supernatants from cultured splenocytes. Tissue homogenates of gastric tissue of different group of mice infected by different combination of *H. pylori* were subjected for analysis of IFN-γ, IL-12 and IL-17 production by ELISA (**a**). Results were expressed as pg/mg total protein. Splenocytes isolated from control and all *H. pylori* infected groups were re-stimulated with or without *H. pylori* WCP (2.5 mg/ml). Supernatants were collected 48 h after stimulation, and secreted cytokines were measured by ELISA (**b**). Results were expressed as pg/ml protein. Gastric tissues extract from controls and *H. pylori* infected mice were analyzed for immunosuppressive cytokines, IL-10 and TGF-β production by ELISA (**c**). IL-10 and TGF-β concentration (pg/mg) were expressed as pg/mg total protein *Error bars* ±SEM ***P < 0.001; **P < 0.01; *P < 0.05; NS, P = not significant versus control; n = 4

Th1 and Th17 cell response plays an important role to mediate inflammation during *H. pylori* induced gastric pathogenesis. Hence, we measured the level of proinflammatory cytokines IFN-γ and IL-17A in *H. pylori* infected mice gastric tissues using ELISA. All mice infected or coinfected with different *H. pylori* strains significantly upregulated gastric IFN-γ compared to control (Fig. 4a) SS1/SS1 infected group secreted highest level of IFN-γ compared to any other groups. In addition, *cag* ^+^ *H. pylori* infection irrespective of sequence of infection significantly induced gastric IFN-γ secretion compared *cag* ^−^ *H. pylori* infection. Even though AM1/SS1 infected mice developed less severe gastric pathology than SS1/AM1, no significant differences in the level of IFN-γ secretion were detected. This observation led us to ask the question whether reduce gastric pathology in AM1/SS1 coinfected mice are due to reduce activation of Th17 response. We found that all mice that were prior infected with *cag* ^+^ *H. pylori* significantly expressed higher level of gastric IL-17A cytokine compared to control (Fig. 4a). However, no significant levels of IL-17A were detected in AM1/AM1 infected or AM1/SS1 coinfected groups. Hence severe gastric damage in *cag* ^+^ infection might be mediated by activation of IL-17 response.

To correlate the Th1 and Th17 cytokines responses in both gastric mucosa and spleen, the splenocytes from the infected mice were stimulated by *H. pylori* WCP in vitro for 48 h. Then, IFN-γ and IL-17A expression level in the supernatants of the cultured splenocytes were measured. Cytokines expression pattern in splenocytes match parallel with gastric tissues in *H. pylori* infected mice (Fig. 4b).

To confirm the involvement of Th1 response in *H. pylori* infection, we further measured the cytokine IL-12 that is responsible for the regulation of Th1 cell response. Our result showed that prior SS1 infection significantly upregulated IL-12 secretion as compared to *cag* ^−^ *H. pylori* infection both in gastric mucosa and spleen (Fig. 4a, b). Highest level of IL-12 was detected in SS1/SS1 infected group while AM1/AM1 exhibited lowest level. No significant difference in the expression of IL-12 was observed in between SS1/AM1 and AM1/SS1 infected groups.

Immunosuppression is mediated by cytokines IL-10 and TGF-β. Hence, we measured the level of IL-10 and TGF-β in *H. pylori* infected mouse gastric tissues. A significant decrease in the level of IL-10 and TGF-β were observed in the AM1/SS1 coinfected mice which correlate with reduced gastric inflammation (Fig. 4c).

### Infiltration of Foxp3^+^ Treg cells in *cag*^+^ strain infected gastric tissue was reduced by earlier *cag*^−^*H. pylori* infection

**Fig. 5:**
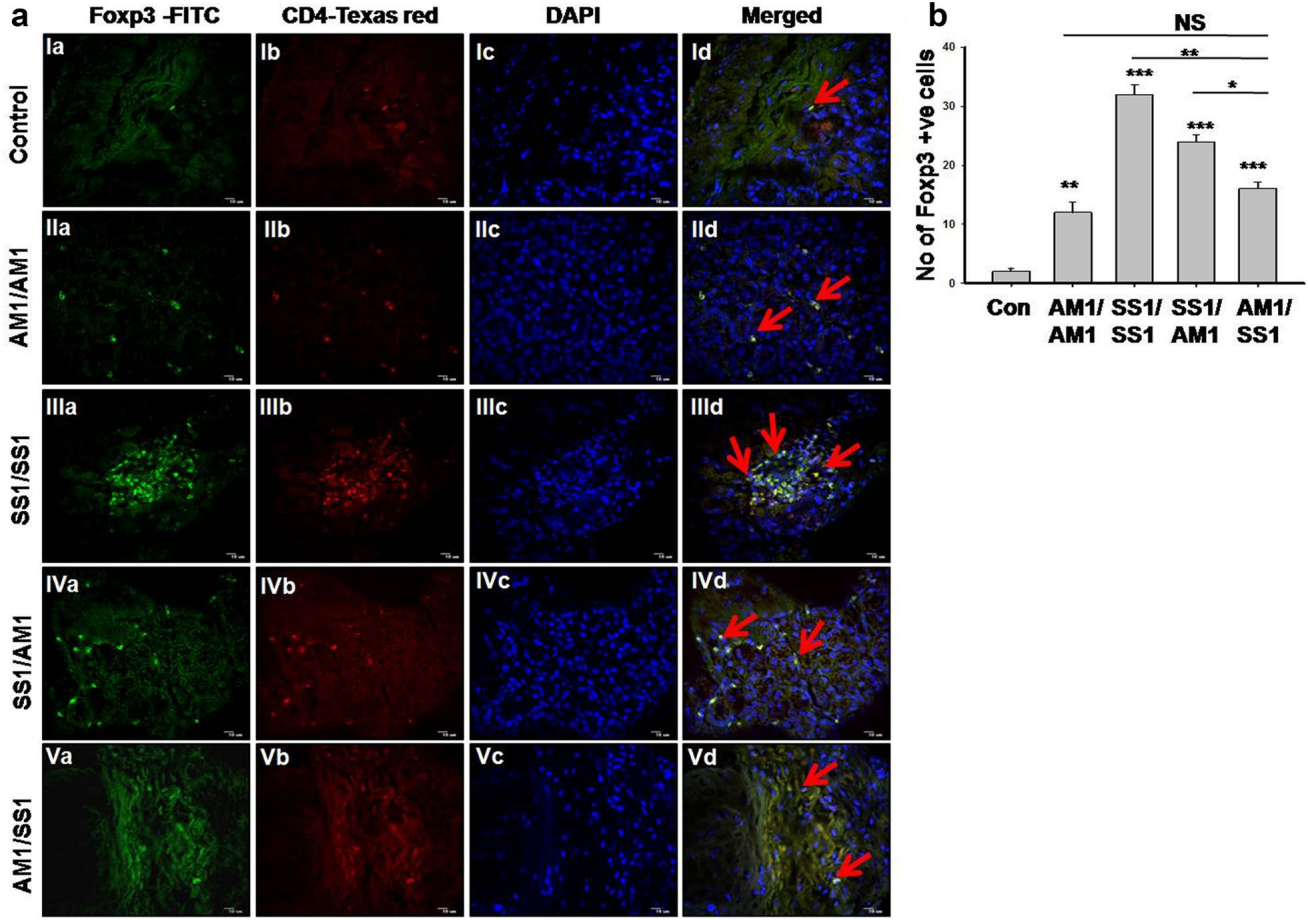
Quantification of Foxp3^+^ Treg cells in *H. pylori* infected mouse gastric tissues. *H. pylori* infected and uninfected gastric tissues were stained for regulatory T cell marker Foxp3 and CD4. Foxp3 were stained with fluorescein isothiocyanate (FITC) (*green*) (*Ia*–*Va*), CD4 were stained with Texas red (*red*) (*Ib*–*Vb*), nuclei were stained with DAPI (*Ic*–*Vc*), while (*Id*–*Vd*) represent their merge pictures **(a)**. Representative immunostaining of regulatory T cells for control (*I*), AM1/AM1 infected (*II*), SS1/SS1 infected (*III*), SS1/AM1-coinfected (*IV*) and AM1/SS1-coinfected (*V*) gastric tissues were observed at 40× magnification. The *red arrow* shows the co-localization of Foxp3 and CD4 in *H. pylori*-infected mouse gastric tissues (*Id*–*Vd*). Average numbers of FoxP3^+^ cells in each tissue sections were assessed from 10 different fields (1 mm^2^). *Bar diagrams* showed the average number of FoxP3^+^ cells present in different groups (**b**). *Error bars* ±SEM ***P < 0.001; **P < 0.01; *P < 0.05; NS, P = not significant versus control

Foxp3 transcription factor is essential for differential development of anti inflammatory Treg cells. Increased numbers of CD4 CD25 Foxp3 regulatory T (Treg) cells were detected in *H. pylori* infected gastric tissues. To understand the immunosuppressive action of Treg cells during *H. pylori* infection or coinfection mediated gastric pathogenesis, we measured the number of Treg cells present in all *H. pylori* infected and coinfected gastric tissues. All groups infected with *H. pylori* showed elevated numbers of gastric Foxp3^+^ cells than control (Fig. 5a, b). Moreover prior *cag* ^+^ *H. pylori* strain infected gastric tissues exhibited higher number of Foxp3^+^ cells than *cag* ^−^ *H. pylori*. Although we did not found any significant differences in the gastric Foxp3^+^ Treg cells between SS1/SS1 infected and SS1/AM1 coinfected mice and between AM1/AM1 infected and AM1/SS1 coinfected mice. In between the coinfected groups AM1/SS1 infected mice exhibited significantly lower gastric Foxp3^+^ cells than SS1/AM1 infected group. Interestingly decrease numbers of FoxP3^+^ Treg cells correlates with the low degree of gastric inflammation in AM1/SS1 infection. Hence, there is a positive association with number of foxp3^+^ Treg cells and gastric inflammation severity in *H. pylori* infection.

### Interplay between inflammatory and immunosuppressive cytokines in *H. pylori* mediated gastric inflammation

**Fig. 6:**
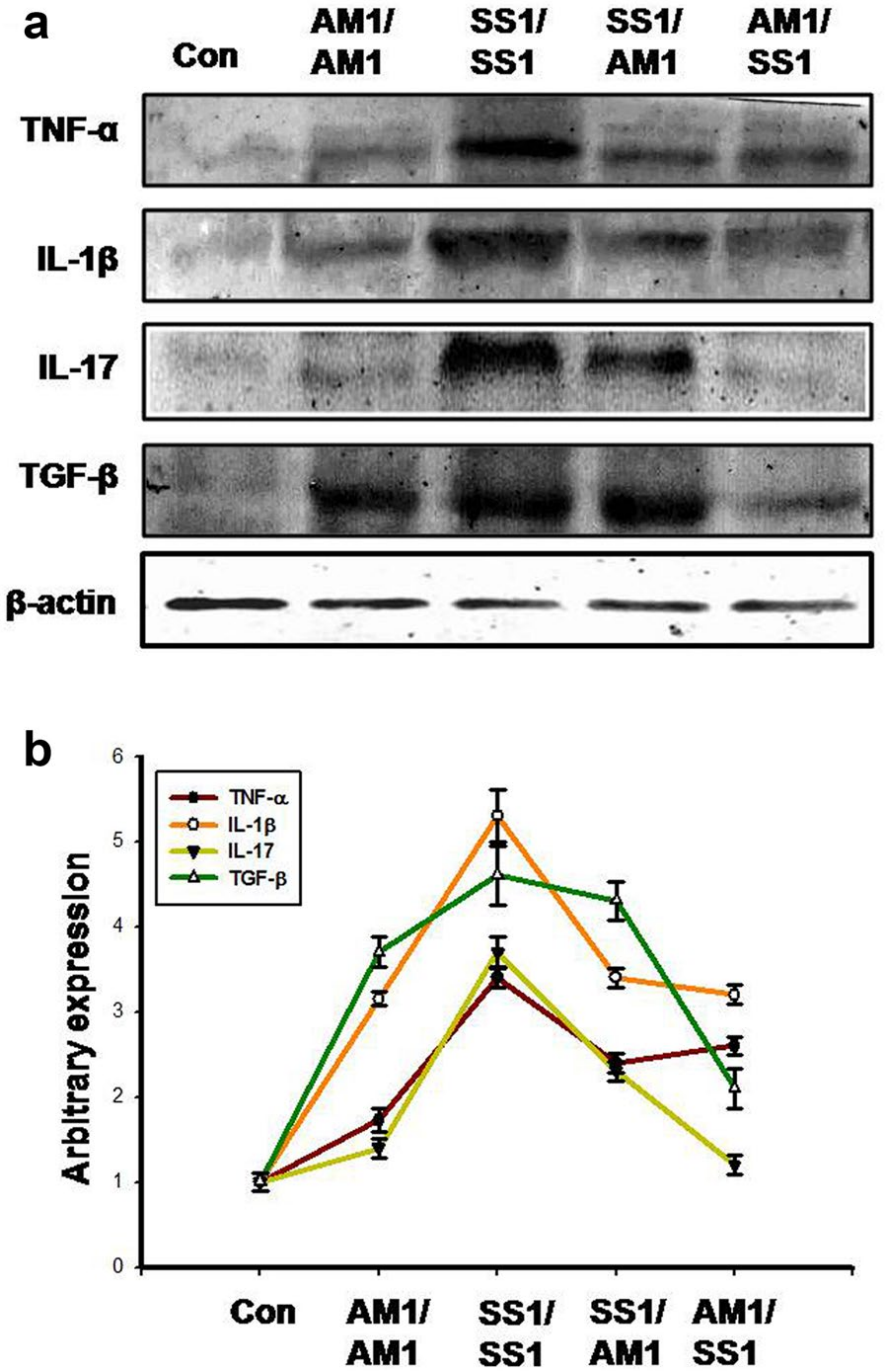
Inter-relation between Th1, Th17 and Treg cytokines in *H. pylori* infected mouse gastric tissues. Different strains of *H. pylori* were orally fed separately or in combination to C57BL/6 mice and they were sacrificed on day 10 postinfection. Control mice were fed with PBS and kept separately under same conditions. Expression of TNF-α, IL-1β, IL-17 and TGF-β in infected mouse gastric tissue homogenates were assessed by Western blotting. Representative Western blots showing the expression of TNF-α, IL-1β, IL-17 and TGF-β in all groups, β-actin served as loading control (**a**). Histographic representation of fold changes at expression level as measured by Lab Image densitometry values (**b**) from the *above blots* and two other representative blots from independent experiments in each case

Balance between inflammatory and immunosuppressive cytokines play important role in various chronic infections including *H. pylori* induced gastric injury. Interplay among Th1, Th17 and Treg cells and their signature cytokines are crucial for *H. pylori* induced pathogenesis. To understand the role of inflammatory (Th17 and Th1) and immunosuppressive (Treg) cytokines in *H. pylori* induced gastric pathology, we compared the level of various cytokines in mice gastric tissues infected or coinfected with different strains of *H. pylori*. We found that *H. pylori* infection increased the expression of Th1 cytokines, TNF-α and IL-1β in mice gastric tissues (Fig. 6). To check the contribution of *cag* PAI on immunomodulation, we compared cytokine expression pattern among different combination of *H. pylori* infected mice gastric tissues. Our result suggested that Th1 cytokines, TNF-α and IL-1β expression increased in *cag* ^+^ infection compared to *cag* ^−^ infection. SS1/SS1 infection exhibited highest level of TNF-α and IL-1β expression as compared to other groups. Although, AM1/SS1 infected mice developed less gastric injury than SS1/AM1, no significant difference in TNF-α and IL-1β cytokines level were observed in between these groups.

We also measured the expression of Th17 cytokine IL-17A in different combination of *H. pylori* strain infected mice gastric tissues. Interestingly, we found that IL-17A expression increased in SS1/SS1 and SS1/AM1 infection, while no significant changes in IL-17A expression were observed in AM/AM1 infected group as compared to control. Surprisingly, AM1/SS1 infected group exhibited no significant increase in IL-17A expression as compared to control. We found a positive correlation between IL-17A expression and severity of gastric inflammation. Expression pattern of immunosuppressive cytokine TGF-β was also measured using Western blotting. Highest level of expression was detected in SS1/SS1 infected group. However, interestingly AM1/SS1 coinfected mice showed decreased TGF-β expression as compared to SS1/SS1 infected and SS1/AM1 coinfected groups. Furthermore, we did not found any significant change in TGF-β expression between AM1/SS1 and AM1/AM1 infected mice gastric tissues. The expression of TGF-β was higher in SS1/AM1 infected mice as compared to AM1/SS1 infected groups.

## Discussion

*Helicobacter pylori* colonization and associated pathology is determined by a combination of pathogen virulence factors and host immune response [5, 6, 38]. *H. pylori* infection induced a robust proinflammatory Th1 and Th17 response that are associated with gastric inflammation, atrophy, epithelial hyperplasia and dysplasia [10, 17, 18, 39]. Moreover, mixed or coinfection of different Helicobacter species/strains determined the outcome of disease severity. In this context, Secka et al. reported that mixed infection with *cag* ^+^ and *cag* ^−^ strains of *H. pylori* lowers disease burden among the Gambian population [31]. Furthermore, coinfection with enterohepatic *Helicobacter* species can reduce *H. pylori* induced gastric pathology in C57BL/6 mice through modulation of gastric Th1 and Th17 responses [30]. In present study we have investigated whether *cag* ^+^ and *cag* ^−^ *H. pylori* coinfection induces gastric mucosal inflammatory response differ from single strain infection. The study also focuses whether coinfection has any modulatory effect on gastric ulcer severity compared to single strain infection. We previously reported that both SS1 and AM1 strains were capable to cause gastric inflammation although the severity of damage was more pronounced in SS1 infection [26]. Although, the functionality of SS1 *cag* gene within mouse gastric tissue is a controversial, but its association with severe gastric inflammation is well established. Our current result suggested that *cag* ^+^ strain (SS1) induced gastric pathology were significantly attenuated in mice that were earlier coinfected with *cag* ^−^ strain (AM1) and associated with modulation of Th17 and Treg cell responses.

It is reported that *H. pylori* infection is associated with elevated Th1 cytokines [10, 40]. Hence, we examined whether reduced gastric inflammation in AM1/SS1 coinfected mice has any correlation with Th1 cytokines level. We found that despite the reduced gastric inflammation pathology in AM1/SS1 infected mice, the expression of inflammatory Th1 cytokines IFN-γ, TNF-α and IL-1β in AM1/SS1 and SS1/AM1 are comparable (Fig. 5). Interestingly, significantly lower level of IL-17 was detected in AM1/SS1 coinfected group than SS1/AM1 coinfected group. Previous studies established the role of proinflammatory Th17 pathway in the development of *H. pylori* induced gastric inflammation in mouse model and human [39, 41]. Yun shi et al. suggested that both Th1 and Th17 cells mediated mucosal inflammation is important in *H. pylori* infection and Th17/IL-17 pathway modulates Th1 cell responses [17]. Th17 cell responses are induced earlier than Th1 cell responses [17], implying that Th17 and Th1 cells promote inflammation differentially. It is known that active type IV secretion system is required for IL-17 secretion [18]. We found that *cag* ^+^ strain infection induced IL-17A secretion in mouse gastric tissues as well as spleen, while *cag* ^−^ infection did not. It seems to us that severe gastric inflammation in SS1/SS1 infected mice were mediated by both Th1 and Th17 responses while AM1/AM1 infection only by Th1 responses. We found that the level of Th1 cytokines IFN-γ, TNF-α and IL-1β in AM1/SS1 infected mice are comparable to SS1/AM1infected mice. In contrast, higher level of IL-17A was detected in SS1/AM1 mice than AM1/SS1 infected mice. Thus our results clearly indicate that attenuated gastric pathology in AM1/SS1 infected group is not due to reduced Th1 responses instead of reduced Th17 responses to AM1/SS1 infection. We conclude that the Th1 cytokine induced by prior AM1 infection particularly IFN-γ could also contribute in part to the downregulation of Th17 response induced by subsequent *cag* ^+^ (SS1) infection because IFN-γ plays an inhibitory role towards Th17 cell activation [42, 43]. Thus, AM1 infection released high level of IFN- γ in the gastric lumen that prevented the activation of Th17 response resulting protection against further *cag* ^+^ infection.

Expression and secretion of different MMPs in *H. pylori* infection have been postulated to be critically involved in the development of gastric ulcer. However, recent evidences suggest that apart from its well studied inflammatory and pathogenic functions, MMPs play a more complex and diverse role in ECM homeostasis, regulation of inflammation, arresting disease progression [22]. Role of cytokines and growth factors in regulation of MMPs expression have been reported earlier under various pathological conditions [22, 44]. IL-17 stimulated gastric epithelial cells to produce MMP9 and 3 that might be important in mediating gastric inflammation. However, a significantly lower level of MMP9 and 3 expressions were detected in AM1/SS1 coinfected mice compared to SS1 alone or SS1/AM1 coinfected group (Fig. 3). In line with our observation, it has been reported that MMP9 expression in the stomach following *H. pylori* infection was significantly reduced when IL-17 is deficient or blocked [17]. Moreover, recombinant IL-17A treatment increased MMP9 expression in vitro [17]. Our results show that the level of IL-17 is significantly increased only in the mouse gastric tissues infected with SS1 strains of *H. pylori*, suggesting that *cag* PAI is required for the induction of IL-17 cytokine, and also indicates that the cells producing MMPs have responded to the increased IL-17 secretions. Our results also suggested that the reduced gastritis in AM1/SS1 infected mice may be is due to reduced activation of Th17/IL-17 pathway and subsequent downregulation of MMP9 and 3 expressions in AM1/SS1 infected group.

It is well established that natural regulatory T (Foxp3^+^ Treg) cells suppress the host inflammatory responses during infection and thereby maintain physiological homeostasis of host immunity [45–47]. Elevated numbers of Treg cells were reported in *H. pylori* positive patients and *H. pylori* infected mice gastric tissues [48–50]. Moreover inhibition of Treg cells function by treatment with monoclonal antibody resulted increased expression of gastric proinflammatory cytokines that lead to severe gastritis in *H. pylori* infected mice [48]. CD4^+^CD25^+^ Treg cells from *H. pylori* positive patients are more potent in the suppression of memory T cell responses [51]. Treg mediated immune suppression is predominantly utilizes IL-10 and TGF-β that currently gain much attention [45, 46]. Previously it has been reported that *H. pylori* induced gastritis was suppressed by adoptive transfer of Treg cells harvested from IL-10-competent C57BL/6 donor mice, demonstrating that IL-10-dependent Treg cells play a crucial role in suppressing *H. pylori*-induced gastric disease [52]. Our results also showed that the number of gastric foxp3^+^ cells as well as gastric IL-10 and TGF-β level were significantly higher in *H. pylori* infected mouse gastric tissues (Figs. 5, 6). While, AM1/SS1 infected mice with attenuated gastritis have fewer foxp3^+^ cells and lower level of gastric IL-10 and TGF-β. Hence we found a positive correlation between severity of gastritis and no of Foxp3^+^ cells as well as IL-10 and TGF-β expression. Previous reports suggested that IL-10 and TGF-β can suppress inflammatory Th17 as well as Th1 responses [53, 54]. So it is reasonable to postulate that prior AM1 infection creating an anti-inflammatory bias to further *H. pylori* infection at the outset of coinfection, with relatively lower demand for Treg cells at more chronic time points because the Th1 and Th17 response to subsequent *H. pylori* infection was suppressed by prior AM1 primed Treg cells.

We hypothesized that dendritic cells exposed to *H. pylori* may promote the preferential differentiation of naïve T cells into Treg cells. Those exposed dendritic cells then assist the differentiation of Treg cells as well as it lost its capability to further induce Th1 and Th17 responses upon subsequent *H. pylori* infection. Thus prior AM1 infected group showed reduced gastritis as its deficiency to induce Th17 response and probably stimulation of an anti-inflammatory bias by accumulation of AM1 sensitized dendritic and Treg cells within the gastric mucosa. In both SS1 and AM1 infection, primed Treg cells generated in gastric mucosa and these Treg cells provide protection against further infection of *H. pylori* by either directly or through cross reactivity. In contrast, prior SS1 infection cause an increase in the level of Th1 and Th17 responses are sufficient to do gastric damage. Irrespective of inhibitory role of SS1 primed Treg cells subsequent SS1 infection enjoy the benefit of existing inflammatory bias for further infection. However, earlier infection with AM1 helps in elicitation of AM1-primed Treg cells as well as less inflammatory bias through reduced secretion of Th1 cytokines. Subsequent infection of SS1 is prevented due to enrichment of AM1 primed Treg cells in the gastric mucosa that might provide protection through creating an anti inflammatory bias as well as by providing an un-hostile environment due to reduced inflammatory bias.

## Conclusions

In summary, we suggest that existed *cag* ^−^ *H. pylori* infection attenuated severe gastric pathology induced by *cag* ^+^ *H. pylori* strain. Reduced gastric pathology is due to an anti-inflammatory bias created by *cag* ^−^ *H. pylori*. Further study is required to elucidate the cascade of interactions between *H. pylori* and mucosal cells, which will provide additional insights into the pathogenesis of *H. pylori.* A better understanding of the nature, regulation and function of the T-cells responses during *H. pylori* coinfection may help to design novel and cost effective strategies through which *H. pylori* induced gastric pathology might be controlled.

## Methods

### Culture of *H. pylori* strains

Two unrelated mouse adapted *H. pylori* strains with different genetic makeup were used: SS1 [34, 35], and AM1 (Indian strain) [26]. SS1 (The Sydney Strain) is widely used as the standard mouse adapted strain for experimental infection. The strain AM1 was isolated from an endoscopic sample of an ulcer patient in Kolkata, India as mixed infections [26]. Both strains of *H. pylori* were grown on brain–heart infusion agar (BHI; Difco Laboratories, Detroit, MI) supplemented with 7% sheep blood, 0.4% isovitalex and the antibiotics amphotericin B (8 µg/ml), trimethoprim (5 µg/ml) and vancomycin (8 µg/ml) (referred to here as BHI agar). The plates were incubated at 37 °C under 5% O_2_, 10% CO_2_ and 85% N_2_. In all experiments, overnight grown cultures on BHI agar plates were used.

### Infection of C57BL/6 mice with *H. pylori*

Male C57BL/6 mice with free access to food and water were obtained from institutional animal house. Experiments were designed to minimize animal suffering and to use the minimum number to obtained valid statistical evaluation. Animal experiments were carried out according to the guidelines of animal ethics committee of the institute. Animals of both control and experimental groups were fasted for 6 h with free access to water. *H. pylori* infection in mice was done using a modification of the Kundu et al. method [34]. Briefly, overnight grown bacterial cultures were harvested in 10 mM phosphate-buffered saline (PBS) and used for inoculation (10^8^ CFU/mouse/inoculation). Mice were divided into five groups (n = 6 in each), first group serves as control was given PBS only. Among the rest 4 groups 2 groups were orogastrically inoculated twice in a period of three days with either AM1 (*cag* ^+^) or SS1 (*cag* ^−^) strain (AM1/AM1 and SS1/SS1) and the rest 2 groups were given criss-cross infection. Criss–cross means infection by *cag* ^+^ strain followed by *cag* ^−^ and vice versa (AM1/SS1 and SS1/AM1). Mice were sacrificed at day 10 after final inoculation (13 days post-primary inoculation).

### Histological analysis

Gastric tissues of control and 10-day infected mice were sectioned for histological studies. The tissue samples were fixed in 10% formalin and embedded in paraffin. The sections (5 µm) were cut using microtome, stained with hematoxylin and eosin [21], and assessed under an Olympus microscope. Images were captured using Camedia software (E-20P 5.0 Megapixel) at original magnification 10 × 10, 20 × 10 and 40 × 10 and processed in Adobe Photoshop version 7.0.

### Tissue extraction

The pyloric part of the gastric mucosa of mice were suspended in PBS containing protease inhibitors, minced and incubated for 10 min at 4 °C. After incubation the suspension was centrifuged at 12,000×*g* for 15 min and the supernatant was collected as PBS extract. The pellet was extracted in the lysis buffer (10 mM Tris–HCl pH 8.0, 150 mM NaCl, and 1% Triton X-100 and protease inhibitors) and centrifuged at 12,000×*g* for 15 min to obtain TX extracts. Tissue extracts were preserved at −80 °C for future studies.

### Serum isolation

Blood samples were isolated from mouse by puncturing the heart followed by incubation for 30 min at room temperature. Serum was isolated from the clotted blood by low centrifugation. Serum sample was mixed with protease inhibitor mixture and stored at −80 °C. Equal volume of serum was used for gelatin zymography.

### Gelatin and casein zymography

For assay of MMP2, 9 and 3 activities, tissue extracts were electrophoresed in 8% SDS–polyacrylamide gel containing 1 mg/ml gelatin or casein (sigma) respectively, under non-reducing conditions. Seventy micrograms proteins were loaded in each lane. The gels were washed twice in 2.5% Triton X-100 (Sigma) and then incubated either in calcium assay buffer or in stromelysin assay buffer at 37 °C. Gels were stained with 0.1% Coomassie blue followed by destaining [21]. Quantification of zymographic-bands were done using Lab-Image software (Kapelan, Gmbh, Germany).

For assay of MMP9 activity in serum, mice serum samples were mixed with 1× nonreducing Laemmli sample loading buffer and were electrophoresed in SDS-8% polyacrylamide gel containing 1 mg/ml gelatin under nonreducing condition. Equal volume of serum samples were loaded in each lane. The gels were washed twice in 2.5% Triton X-100 and incubated in calcium assay buffer at 37 °C. Gels were then stained with 0.1% Coomassie Brilliant Blue stain followed by destaining. The zone of gelatinolytic activities appeared as negative staining. Quantification of zymographic bands were performed by densitometric analysis using Lab Image software (Kapelan Gmbh, Germany).

### Measurement of cytokines by ELISA

*Helicobacter pylori* infected and uninfected mice gastric tissues were homogenized in 1 ml sterile PBS, and centrifuged. The supernatants were analyzed for IFN-γ, IL-12, IL-17, IL-10 and TGF-β using sandwich ELISA kits (eBioscience, San Diego, CA) according to the manufacturer’s instruction. Total protein was measured by the Lowry method. The cytokines concentrations in gastric tissue extracts were expressed as picograms per milligrams of total protein [17].

### Splenocytes culture and cytokine measurement

*Helicobacter pylori* infected and uninfected mice spleens were passed through meshed steel sieve to obtain single-cell suspension of splenocytes. Splenocytes (1.6 × 10^6^ cells/ml) were cultured in RPMI 1640 medium with or without *H. pylori* whole cell protein (WCP) (2.5 μg/ml).The production of IFN-γ, IL-12 and IL-17 in the supernatants was measured by sandwich ELISA (eBioscience, San Diego, CA) after 48 h of splenocytes culture [17].

### Immunofluoresence

For Immunofluorescence study, the tissue samples were fixed in 4% paraformaldehyde solution for 48 h, dehydrated in ascending alcohol series [36]. It was embedded in paraffin wax and sectioned at 5 mm thickness using a microtome. The sections were deparaffinized with xylene followed by rehydration with descending alcohol series. Antigen retrieval was performed by trypsin (0.05% trypsin, 0.1% CaCl2) and blocking was performed using 5% BSA in TBS (20 mM Tris–HCl, pH 7.4 containing 150 mM NaCl) for 2 h at room temperature followed by the incubation over night at 4 °C in primary antibody solution (1:200 dilutions in TBS with 1% BSA) in a humid chamber. The tissue sections were washed four times with TBST (20 mM Tris HCl, pH 7.4 containing 150 mM NaCl and 0.025% Triton X-100) followed by incubation with fluorescein isothiocyanate and Texas Red-conjugated secondary antibody (Santa Cruz Biotechnology) solution. Then the tissue sections were washed four times with TBST followed by nuclear staining with DAPI. The images were observed in confocal microscopy. Images at X40 magnification were captured using Andor iQ 2.7 software (Andor spinning dise confocal microscope, Belfast, Ireland) and processed under Adobe Photoshop version 7.0 (Adobe Systems, San Jose, CA).

### Western blotting

Tissue extracts (120 μg) were resolved by 10% reducing SDS–polyacrylamide gel electrophoresis and transferred to nitrocellulose membranes [37]. The membranes were blocked for 2 h at room temperature in 3% bovine serum albumin solution in 20 mM Tris–HCl, pH 7.4 containing 150 mM NaCl and 0.02% Tween 20 (TBST) followed by overnight incubation at 4 °C with 1:500 polyclonal anti-MMP9 (sc-6841, Santa Cruz Biotechnology), MMP3 (sc-6839, Santa Cruz Biotechnology), TNF-α (sc-1351, Santa Cruz Biotechnology), IL-1β (sc-7884, Santa Cruz Biotechnology), IL-17 (sc-374218, Santa Cruz Biotechnology), TGF-β (sc-7892, Santa Cruz Biotechnology,) and β-actin (4967S, cell signalling technology, MA, USA) antibodies. The membranes were washed four times with TBST and then incubated with their respective alkaline phosphatase-conjugated secondary antibody (Santa Cruz Biotechnology) (1:2000) for 1.5 h. The bands were visualized using 5-bromo-4-chloro-3-indolyl phosphate/nitro blue tetrazolium substrate solution (Sigma).

### Statistical analysis

Densitometry data are fitted using Sigma Plot. Data are presented as the mean ± SE. Statistical analysis was performed using the Student’s *t* test. P value less than 0.05 were considered as significant.

## Abbreviations

MMP: matrix metalloproteinase
ECM: extracellular matrix
PAI: pathogenicity island
BHI: brain heart infusion agar
FBS: fetal bovine serum
IL: interleukin
PBS: phosphate-buffered saline
TNF: tumor necrosis factor
AGS: gastric adenocarcinoma
Treg: T-regulatory
TGF: transforming growth factor
